# Meat Eaters

**Published:** 1892-05

**Authors:** 


### MEAT EATERS.

Many races of men live entirely on animal food, and these are the most hardy, and, from all I have been able to gather on the subject, the most free from diseases of all kinds. Sir Francis Head says of the Pampas Indians : They are all horsemen, or, rather, pass their lives on horseback. In spite of the climate, which is burning hot in summer and freezing in winter, these brave men, who have never yet been subdued, are entirely naked and have not even a covering for their head. They live together in tribes, each of which is governed by a cacique, but they have no fixed place of residence. Where the pasture is good, there are they to be found until it is consumed by their horses, and they instantly move to a more verdant spot. They have neither bread, fruit, nor vegetables, but they subsist entirely upon the flesh of their mares.

Describing the effect upon himself of this diet, Sir Francis says: After I had been riding three or four months, and had lived on beef and water, I found myself in a condition which I can only describe by saying that I felt as if no exertion would kill me, although I constantly arrived so completely exhausted that I could not speak; yet a few hours sleep upon my saddle on the ground always so completely restored me that for a week I could daily be upon my horse before sunrise, could ride two or three hours after sunset, and have really tired ten or twelve horses a day. This will explain the immense distances which people in South America are said to ride, which I am confident could only be done on beef and water. The Guachos of the Argentine Republic live entirely on roast beef and salt, scarcely ever tasting farinaceous or other vegetable food, and their sole beverage is mate or Paraguay tea taken without sugar.

